# Comparison of assistance preferences of older adults with different functional dependence levels on domestic tasks performed by robots

**DOI:** 10.1186/s12877-023-04567-w

**Published:** 2024-01-13

**Authors:** Linda Yin-king Lee, Chun-kit Yeung, Chun-wa Choi, Man-nga Leung, Shing-yan Lui, Wing-yi Tam, Ka-yi Tang, Chun-san Wong, Yuen-shan Wong, Cheuk-yi Yau, Tik-ling Yeung, Joseph Kok-long Lee, Debby Lee-kuen Chui

**Affiliations:** 1 School of Nursing and Health Studies, Hong Kong Metropolitan University, Hong Kong, China; 2 School of Nursing, St. Teresa’s Hospital, Hong Kong, China

**Keywords:** Domestic tasks, Assistance, Robot, Functional dependence level

## Abstract

**Background:**

Robots have the potential to assist older adults in their home-based daily living tasks. Previous studies indicated that older adults generally accept robot assistance. However, the preferences of older adults with different functional dependence levels are lacking. These older adults encounter varying levels of difficulty in daily living and may have distinct preferences for robot assistance. This study aimed to describe and compare the preferences for robot assistance on domestic tasks in older adults with different functional dependence levels.

**Methods:**

This cross-sectional descriptive study recruited a convenience sample of 385 older adults in Hong Kong. They were categorized as independent, partially dependent, and dependent using the Katz Index of Independence in Activities of Daily Living. Their preferences for robot assistance on a list of 48 domestic tasks under six categories were assessed through the Assistance Preference Checklist. Differences in preferences between the three groups were compared using one-way ANOVA test.

**Results:**

Findings revealed the differences and similarities in preferences between participants with different dependence levels. In most domestic tasks under the personal care category, dependent and partially dependent older adults reported a significantly lower preferences for human assistance or a higher preferences for robot assistance (*p* < 0.001), compared with the independent ones. The effect size varied from medium to large (eta squared = 0.07 to 0.52). However, participants, regardless of functional dependence levels, preferred human to assist in some domestic tasks under the health and leisure activities category and preferred robot to assist in most of the domestic tasks under the chores, information management, and manipulating objects category.

**Conclusions:**

Older adults with different levels of functional dependence exhibit different preferences for robotic assistance. To effectively use robots and assist older adults as they age, the specific preferences of older adults must be considered before designing and introducing robots in domestic care.

## Background

Aging is often accompanied by changes in physical, perceptual, and cognitive abilities in older adults. While these changes vary among older adults, they tend to lean towards decline and may result in increased functional dependence for certain older adults [1]. It is likely that older adults who are living in their homes experience varying levels of difficulty in performing daily tasks [2, 3].

The Centers for Disease Control and Prevention of the United States defined aging in place as: “the ability to live in one's own home and community safely, independently, and comfortably, regardless of age, income, or ability level” [4]. This definition highlights that functional dependence should not prevent an older adult from living in their own home. In order to support older adults to age in place, it is essential to ensure they receive suitable and sufficient domestic assistance [1, 5]. However, the supply of care cannot keep pace with the increasing assistance demand from the older population [1, 2]. With the advancement of technology, efforts have been made to introduce robots to assist older adults [6].

Introducing a robot into the home is not simple. Understanding older adults’ preferences for robot assistance is an initial and important step. It allows us to match their preferences to the specific robots and increases acceptance [7, 8]. Due to the difference levels of functional dependence among older adults, they require different types of assistance. Moreover, they may have different preferences for robot assistance. However, there are limited studies conducted in this specific area.

### Use of robots in eldercare

There are various types of service robots utilized in eldercare. They can be used in personal and professional settings. Social robots are designed to interact with users in a social and engaging manner, provide companionship, and promote psychosocial well-being [9]. Assistive robots assist humans to perform physical tasks or activities [10]. Socially assistive robots help users to build social behavior skills through social rather than physical interaction [11]. This research refers all these types of service robots as robots in general.

### Preferences for assistance on domestic tasks from robots in older adults

Older adults prefer robot to assist in information management [12] and object manipulation [1, 13, 14]. In contrast, they do not prefer robots to assist in personal care [1, 13, 14]. Their preferences regarding leisure activities, health, and chores did not reveal a specific pattern [1, 12]. It is important to note that previous studies have certain limitations, such as using a small sample size (range from 21 to 32) [1, 8, 13] and using volunteers as participants [8].

Previous studies were conducted on older adults with different health characteristics, such as functionally independent [1], with memory complaints [8], with cognitive impairments [15], or experiencing difficulties in instrumental activities of daily living [14]. One study was conducted on community-dwelling older adults without describing their functional dependence levels [12]. Another study was conducted on older adults with varying capabilities but did not separately evaluate their preferences [13]. To date, there have been no published studies that comprehensively describe and compare the assistance preferences on domestic tasks from robots in older adults with different levels of functional dependence. Older adults who are functionally independent may not think they have significant impairments. They may consider technology useful only for the older adults with greater levels of frailty than themselves [8]. In contrast, older adults who are functionally dependent face a higher level of difficulty in daily living and may prefer more practical support from robots in daily tasks [2, 8]. Since there is no evidence to confirm these assumptions, the research question of this study was formulated as follow: Are there any differences in assistance preferences on domestic tasks from robots in older adults who fall into the categories of independent, partially dependent, and dependent? Having such an understanding enables us to gain a fuller picture of older adults’ preferences and fill the knowledge gap.

Moreover, knowing the specific preferences of older adults before the introduction of robots is essential because preferences for robot assistance is a predictor to accepting robots, whereas acceptance increases the likelihood of using robots [1, 16]. If their preferences are not addressed, older adults are likely to leave robots aside or even suffer from the negative effects of unsuitable technology [8].

## Methods

### Aim

This study aimed to describe and compare the assistance preferences on domestic tasks from robots in older adults with different functional dependence levels.

### Design

This study adopted a cross-sectional descriptive design. This design is effective in describing the characteristics and making inferences about possible relationships that exist in a population without manipulating it. This study is part of a major study which investigated the preferences for domestic assistance from robots in older adults.

### Sampling

A convenience sample of 385 older adults were recruited in Hong Kong. Older adults aged 65 years old or above and had no prior experience with robots were eligible to participate. Moreover, they were not given any examples of robots capable of providing assistance in a domestic setting. This approach aimed to lessen the potential influence of robotic experience on the preferences of older adults. Those who could not speak Cantonese (a common language in Hong Kong) or were diagnosed with cognitive impairment were not eligible to participate.

Sampling was conducted in public areas in the three main regions of Hong Kong (Hong Kong Island, Kowloon, New Territories) on weekdays and weekends, and from 9 am to 9 pm, thereby increasing the heterogeneity of the sample. Older adults were approached and invited to participate. The sample size was determined in accordance with the Cochran Formula for a population of 1,451,500 older adults at 65 years or above [17], with a confidence level of 95%, and a margin of error of 5%.

### Data collection

The Katz Index of Independence in Activities of Daily Living (Katz ADL) was used to assess participants’ functional dependence level. The Katz ADL comprises six items assessing an older adult’s ability to perform activities of daily living, including bathing, dressing, toileting, transferring, continence, and feeding. Each item can be rated as 1 (independence) or 0 (dependence). By summing the score of the six items, a total score can be calculated. Based on the calculated total score, older adults can be classified into three levels of dependence: independent (6 points); partially dependent (3–5 points); dependent (≤ 2 points) [18]. The Katz ADL is valid and reliable. It showed satisfactory construct validity, which was supported by the known group technique. Patients with dementia reported a lower score in Katz ADL than patients without dementia [19]. It also showed satisfactory internal consistency (Cronbach’s alpha = 0.97 and 0.84) (Ferretti-Rebustini et al., 2015; Arik et al., 2015) [18, 19] and excellent test–retest reliability (Intraclass Correlation Coefficient = 1.000) [19, 20].

The English version of Katz ADL was translated to Chinese through forward and backward translation. A 20-person expert panel (one academic specialized on instrument validation, four academics specialized on translation, 15 nurses specialized on geriatrics) evaluated the semantic equivalence between the two language versions and confirmed that the Katz ADL (Chinese version) was appropriately translated. A 3-people expert panel (one academic specialized on instrument validation and two nurses specialized on geriatrics) was established to evaluate the content validity of the checklist and concluded that the Katz ADL (Chinese version) showed good content validity. The Content Validity Index at the item level and scale level was 1.00. To evaluate stability, an additional group of 30 older adults, distinct from the current sample, were invited to complete the checklist twice at a 1-week interval. The Katz ADL (Chinese version) showed good stability. The test–retest reliability coefficient was 0.85 [21].

The Assistance Preference Checklist was used to assess participants’ preferences to receive robot assistance at home. It is a 48-item checklist which comprises 48 home-based tasks that are considered by older adults as important for fulfilling their general needs and maintaining their homes. The tasks are presented under six categories: personal care, leisure activities, health, chores, information management, and manipulating objects. Participants were instructed to imagine having a robot that can perform domestic tasks to the human level. They rated their preference for assistance in each task on a 5-point Likert scale (1 = only a human, 2 = prefer a human, 3 = no preference, 4 = prefer a robot, 5 = only a robot). The Assistance Preference Checklist demonstrated excellent internal consistency (Cronbach’s alpha = 0.97) [1].

The English version of Assistance Preference Checklist was translated to Chinese and tested using the above-mentioned methods. The 20-person expert panel confirmed that the Assistance Preference Checklist (Chinese version) was appropriately translated. The 3-people expert panel evaluated the content validity of the checklist and supported its good content validity. The Content Validity Index at the item level and scale level was 1.00. By testing it on 50 older adults, the Assistance Preference Checklist (Chinese version) showed excellent internal consistency with a Cronbach’s alpha of 0.95 [22].

The questionnaires were administered by the researchers through face-to-face interviews. This method overcame participants’ difficulty in reading the questions due to impaired visual ability or illiteracy. A pilot study was conducted on 30 older adults to test the data collection procedures. Overall, the older adults needed 20 min to answers all the questions without difficulty.

### Data analysis

Data were normality tested using normal probability plots. All variables were normally distributed and fit for parametric analysis. Descriptive analyses were done for all variables. One-way analysis of variance (ANOVA) was used to compare the difference in preferences between older adults with different functional dependence levels for each domestic task. Tukey’s test was used to confirm the differences between groups. To compensate the chance of making an inflated Type I error in simultaneous multiple comparisons, a Bonferroni correction with *p* < 0.001 was adopted. The adjusted *p* value was calculated by dividing the required overall alpha level of 0.05 by the number of comparison tests to be conducted (i.e., 48).

### Ethical considerations

This study obtained ethical approval from the Research Ethics Committee of the School of Nursing and Health Studies of the Hong Kong Metropolitan University (2021–04). All the participants were informed of the background, aims, and nature of the study. Moreover, they were informed of their rights of participation and withdrawal. They knew that they would not receive any remuneration for participation. All participants provided verbal informed consent which was approved by the Research Ethics Committee of the School of Nursing and Health Studies of the Hong Kong Metropolitan University (2021–04). Waiving of written informed consent for study participation was approved by the above-named Research Ethics Committee. All research methods were carried out in accordance with relevant guidelines and regulations.

## Results

### Participants’ characteristics

A total of 408 questionnaires were administered. Among them, 385 questionnaires were considered valid with no missing data. Participants’ age ranged from 65 to 99 (mean = 74.54, standard deviation = 6.78). Among them, 208 (54.0%) were male and 177 (46.0%) were female. Regarding their marital status, 18 (4.7%) were unmarried, 223 (57.9%) were married, 110 (28.6%) were widowed, and 34 (8.8%) were divorced. Regarding their functional dependency level, 197 (51.2%) were independent, 98 (25.5%) were partially dependent, and 90 (23.4%) were dependent.

### Participants’ preferences for assistance on domestic tasks

**Fig. 1:**
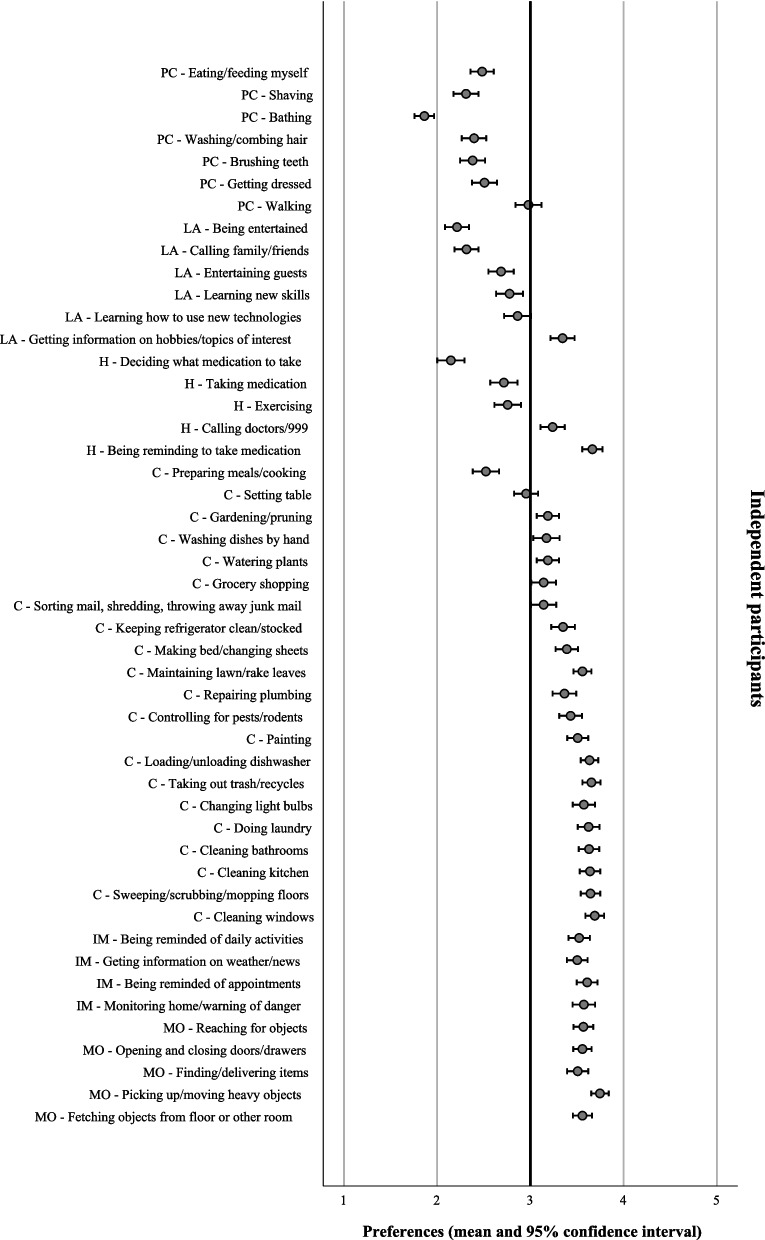
Assistance preferences on domestic tasks from robots in independent older adults (*n* = 197). PC: Personal care; LA: Leisure activities; H: Health; C: Chores; IM: Information management; MO: Manipulating objects

**Fig. 2:**
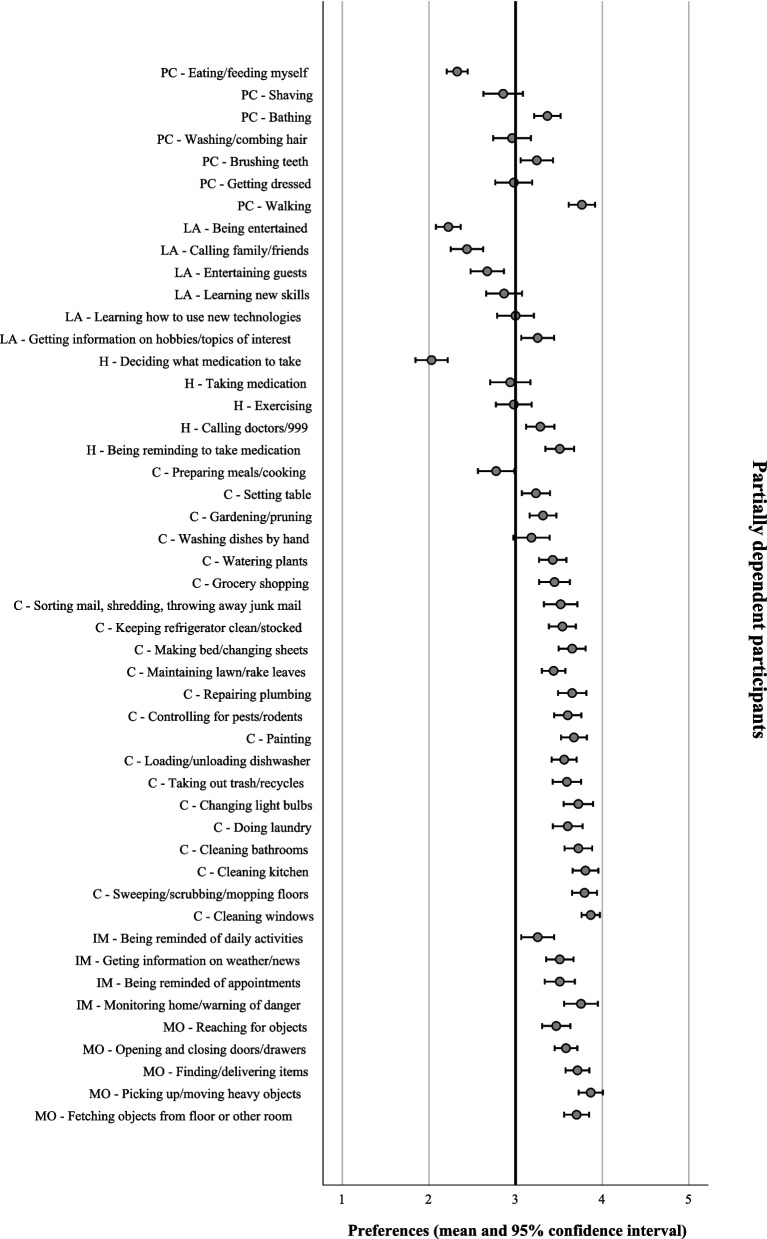
Assistance preferences on domestic tasks from robots in partially dependent older adults (*n* = 98). PC: Personal care; LA: Leisure activities; H: Health; C: Chores; IM: Information management; MO: Manipulating objects

**Fig. 3:**
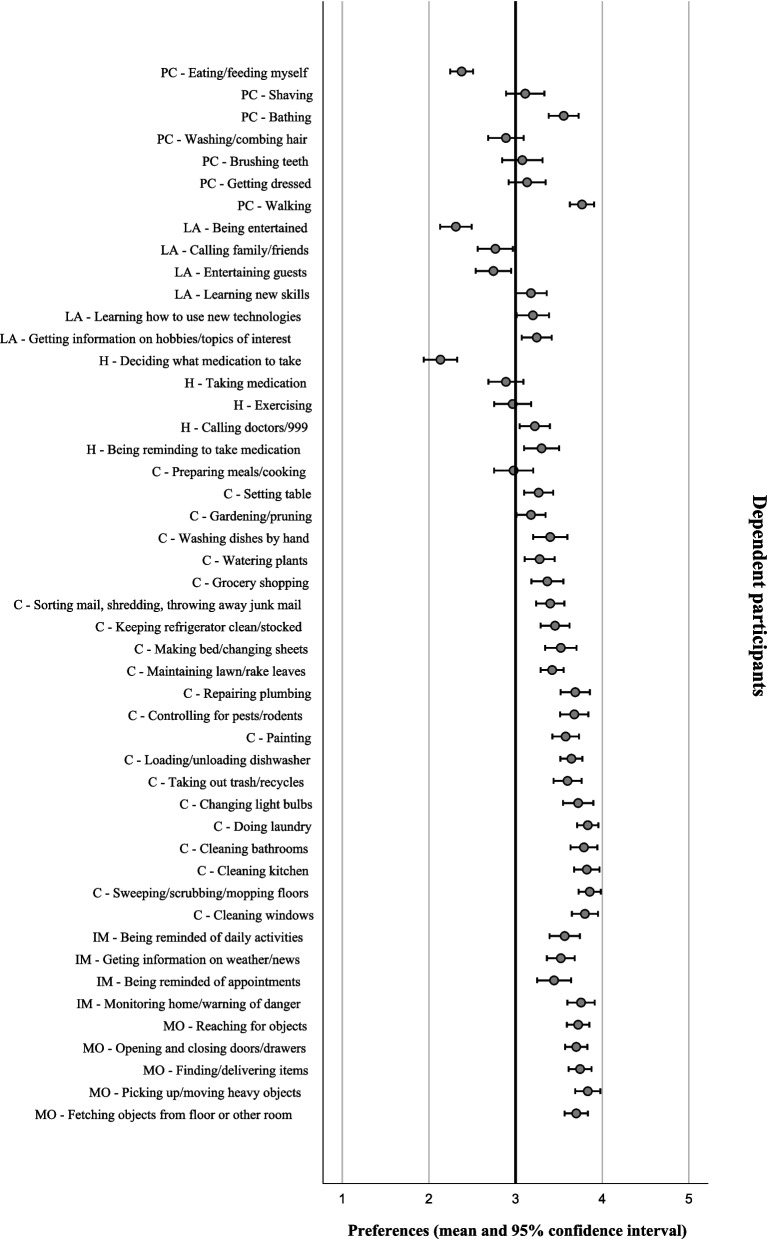
Assistance preferences on domestic tasks from robots in dependent older adults (*n* = 90). PC: Personal care; LA: Leisure activities; H: Health; C: Chores; IM: Information management; MO: Manipulating objects

**Fig. 4:**
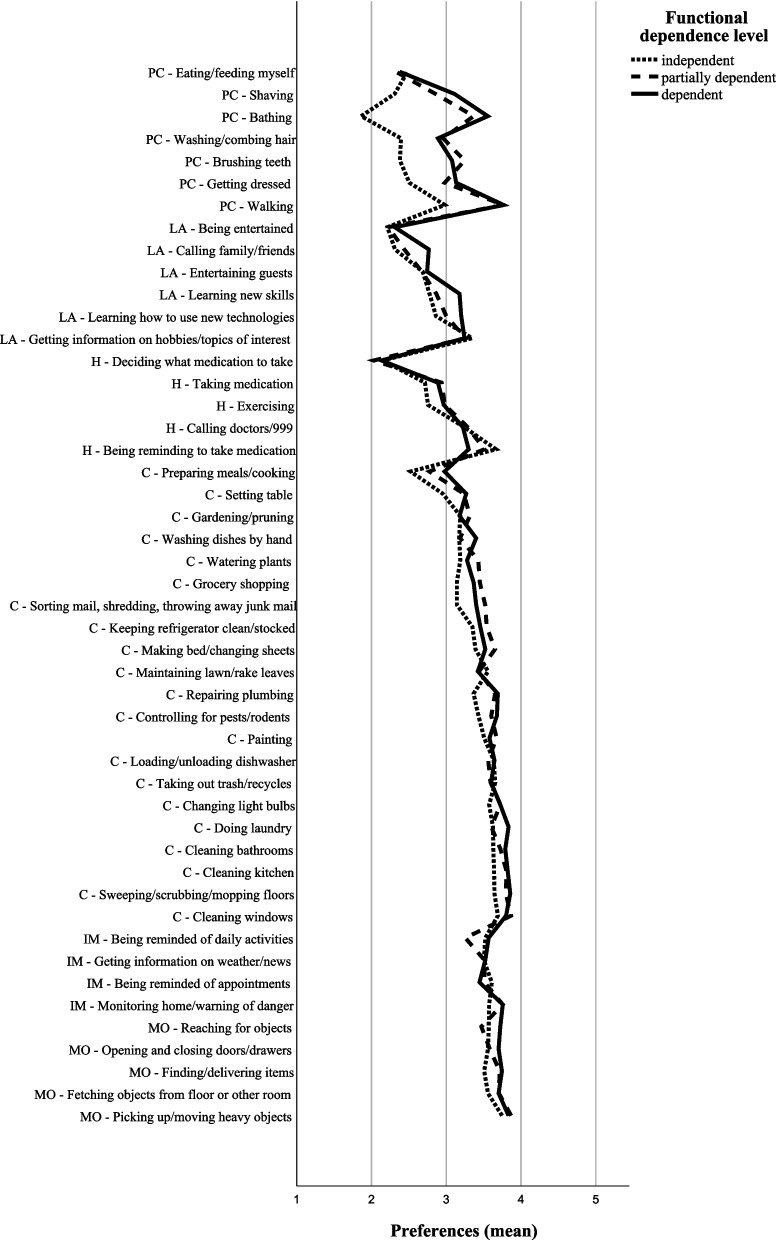
Assistance preferences on domestic tasks from robots in older adults with different functional dependence level (*n* = 385). PC: Personal care; LA: Leisure activities; H: Health; C: Chores; IM: Information management; MO: Manipulating objects

With reference to the mean scores which represented participants’ preferences, the collective group of participants revealed a preference for human assistance in 15 domestic tasks (mean < 3) and robot assistance in 33 domestic tasks (mean > 3) (Table 1). Notably, participants with different functional dependence levels showed different preferences for assistance. They demonstrated obvious differences in preferences in the domestic tasks under the personal care and leisure activities category. However, they demonstrated similar preferences in the domestic tasks under the health, chores, information management, and manipulating objects category (Figs. 1, 2, 3 and 4).

**Table 1:** Assistance preferences on domestic tasks from robots in older adults (*n* = 385)

**Domestic category **	**Domestic task**	**Preferences ** **(Mean** **±** **SD**^**a**^**)**
Personal care	Eating/feeding myself	2.42 ± 0.77
	Shaving	2.64 ± 1.08
	Bathing	2.64 ± 1.11
	Washing/combing hair	2.65 ± 1.02
	Brushing teeth	2.76 ± 1.06
	Getting dressed	2.77 ± 1.04
	Walking	3.36 ± 0.95
Leisure activities	Being entertained	2.24 ± 0.86
	Calling family/friends	2.45 ± 0.95
	Entertaining guests	2.70 ± 0.97
	Learning new skills	2.89 ± 1.00
	Learning how to use new technologies	2.98 ± 1.01
	Getting information on hobbies/topics of interest	3.30 ± 0.91
Health	Deciding what medication to take	2.11 ± 0.10
	Taking medicine	2.81 ± 1.06
	Exercising	2.86 ± 1.02
	Calling doctors/911 (or 999 in Hong Kong)	3.25 ± 0.88
	Being reminded to take medicine	3.54 ± 0.84
Chores	Preparing meals/cooking	2.69 ± 1.05
	Setting the table	3.10 ± 0.88
	Gardening/pruning	3.22 ± 0.82
	Washing dishes by hand	3.23 ± 1.01
	Watering plants	3.27 ± 0.83
	Grocery shopping	3.27 ± 0.92
	Sorting mail, shredding, throwing away junk mail	3.30 ± 0.94
	Keeping refrigerator clean/stocked	3.42 ± 0.85
	Making bed/changing sheets	3.49 ± 0.84
	Maintaining lawn/raking leaves	3.50 ± 0.67
	Repairing plumbing	3.51 ± 0.87
	Controlling for pests/rodents	3.53 ± 0.83
	Painting	3.57 ± 0.77
	Loading/unloading dishwasher	3.62 ± 0.67
	Taking out trash/recyclables	3.63 ± 0.75
	Changing light bulbs	3.65 ± 0.85
	Doing laundry	3.67 ± 0.79
	Cleaning bathrooms	3.69 ± 0.78
	Cleaning kitchen	3.72 ± 0.76
	Sweeping/scrubbing/mopping	3.73 ± 0.71
	Cleaning windows	3.76 ± 0.68
Information management	Being reminded of daily activities	3.46 ± 0.86
	Getting information on weather/news	3.51 ± 0.78
	Being reminded of appointments	3.55 ± 0.85
	Monitoring home/warning about dangers	3.66 ± 0.87
Manipulating objects	Reaching for objects	3.58 ± 0.74
	Opening and closing doors/drawers	3.60 ± 0.67
	Finding/delivering items	3.62 ± 0.75
	Fetching objects from floor or other room	3.63 ± 0.71
	Picking up/moving heavy objects	3.80 ± 0.68

^a^*SD* Standard deviation

#### Personal care category

Independent participants preferred humans to assist all the tasks in the personal care category. Comparatively, the partially dependent and dependent participants reported a higher mean score in their preferences. They preferred some tasks to be assisted by humans but other tasks to be assisted by robots. One-way ANOVA reported a significant difference in preference between participants with different functional dependence level in six out of seven tasks (*p* < 0.001). The effect size, as calculated using eta squared, varied from 0.07 to 0.52, indicating a medium to large effect [23]. Post Hoc Test (Turkey HSD) reviewed a significant difference in preference between the independent and the partially dependent participants and between the independent and the dependent participants (*p* < 0.001). Comparing with the independent participants, the partially dependent and dependent participants reported either a less favorable preference for humans (mean < 3) or a more favorable preference for robots (mean > 3) to assist in shaving, bathing, getting dressed, walking, and brushing teeth (Table 2).

**Table 2:** Assistance preferences on domestic tasks (under the personal care category) from robots in older adults with different functional dependence level

**Domestic task**	**Preferences (Mean** **±** **SD**^**a**^**)**			***F***	***p***	**Eta squared**
	**Independent ** **(*n* = 197)**	**Independent ** **(*n* = 197)**	**Independent ** **(*n* = 197)**			
Shaving	2.31 ± 0.96	2.86 ± 1.14	3.11 ± 1.05	21.736	< 0.001*	0.10
Bathing	1.86 ± 0.75	3.37 ± 0.77	3.56 ± 0.82	207.830	< 0.001*	0.52
Washing/combing hair	2.40 ± 0.93	2.96 ± 1.08	2.89 ± 0.98	14.053	< 0.001*	0.07
Eating/feeding myself	2.48 ± 0.89	2.33 ± 0.61	2.38 ± 0.63	1.504	0.224	-
Getting dressed	2.51 ± 0.96	2.98 ± 1.07	3.13 ± 1.02	14.887	< 0.001*	0.07
Walking	2.98 ± 1.00	3.77 ± 0.76	3.77 ± 0.67	39.220	< 0.001*	0.17
Brushing teeth	2.38 ± 0.96	3.24 ± 0.93	3.08 ± 1.11	30.961	< 0.001*	0.12
**Post hoc analysis (Tukey HSD)**						
**Domestic task **	**Comparing groups**		**Mean difference**	**Standard error **	***p***	
Shaving	Independent versus partially dependent		-0.55	0.127	< 0.001*	
	Independent versus dependent		-0.80	0.131	< 0.001*	
	Partially dependent versus dependent		-0.25	0.150	0.211	
Bathing	Independent versus partially dependent		-1.50	0.095	< 0.001*	
	Independent versus dependent		-1.69	0.098	< 0.001*	
	Partially dependent versus dependent		-0.19	0.112	0.216	
Washing/combing hair	Independent versus partially dependent		-0.56	0.122	< 0.001*	
	Independent versus dependent		-0.49	0.125	< 0.001*	
	Partially dependent versus dependent		0.07	0.144	0.876	
Getting dressed	Independent versus partially dependent		-0.47	0.124	< 0.001*	
	Independent versus dependent		-0.63	0.127	< 0.001*	
	Partially dependent versus dependent		-0.15	0.146	0.544	
Walking	Independent versus partially dependent		-0.79	0.108	< 0.001*	
	Independent versus dependent		-0.79	0.111	< 0.001*	
	Partially dependent versus dependent		-0.01	0.127	1.000	
Brushing teeth	Independent versus partially dependent		-0.86	0.122	< 0.001*	
	Independent versus dependent		-0.70	0.126	< 0.001*	
	Partially dependent versus dependent		0.17	0.144	0.479	

^a^*SD* Standard deviation

*p < 0.001. A Bonferroni correction with a significance level of *p* < 0.001 was applied

#### Leisure activities category

The three groups of participants preferred some tasks to be assisted by humans but other tasks to be assisted by robots. One-way ANOVA reported a significant difference in preference between participants with different functional dependence level in the task calling family/friends (*p* < 0.001). The effect size, as calculated using eta squared, was 0.04, indicating a small effect [23]. Post Hoc Test (Turkey HSD) reviewed a significant difference in preference between the independent and dependent participants (*p* < 0.001). Despite that both groups preferred humans to assist in calling family/friends, the dependent participants reported a less favorable preference for humans (Table 3).

**Table 3:** Assistance preferences on domestic tasks (under the leisure activities category) from robots in older adults with different functional dependence level

**Domestic task**	**Preferences (Mean** **±** **SD**^**a**^**)**			***F***	***p***	**Eta squared**
	**Independent ** **(*n* = 197)**	**Independent ** **(*n* = 197)**	**Independent ** **(*n* = 197)**			
Entertaining guests	2.69 ± 0.97	2.67 ± 0.96	2.74 ± 0.98	0.152	0.859	-
Being entertained	2.21 ± 0.92	2.22 ± 0.71	2.31 ± 0.87	0.421	0.657	-
Calling family/friends	2.31 ± 0.91	2.44 ± 0.93	2.77 ± 0.97	7.301	< 0.001*	0.04
Learning new skills	2.78 ± 1.03	2.87 ± 1.03	3.18 ± 0.87	5.087	0.007	-
Getting information on hobbies/topics of interest	3.35 ± 0.92	3.26 ± 0.95	3.24 ± 0.83	0.534	0.587	-
Learning how to use new technologies	2.86 ± 1.03	3.00 ± 1.06	3.20 ± 0.89	3.511	0.031	-
**Post hoc analysis (Tukey HSD)**						
**Domestic task **	**Comparing groups**		**Mean difference**	**Standard error **	***p***	
Calling family/friends	Independent versus partially dependent		-0.12	0.115	0.528	
	Independent versus dependent		-0.45	0.118	<0.001*	
	Partially dependent versus dependent		-0.33	0.136	0.043	

^a^*SD* Standard deviation

*p < 0.001. A Bonferroni correction with a significance level of *p* < 0.001 was applied

#### Health category

Consistently, the three groups preferred a human to assist in deciding what medication to take, taking medicine, and exercising. Concurrently, they preferred a robot to assist in calling doctors/911 (or 999 in Hong Kong) and being reminded to take medicine. One-way ANOVA did not reveal any significant difference in preference between participants with different functional dependence levels (Table 4).

**Table 4:** Assistance preferences on domestic tasks (under the health category) from robots in older adults with different functional dependence level

**Domestic task**	**Preferences (Mean** **±** **SD**^**a**^**)**			***F***	***p***
	**Independent ** **(*n* = 197)**	**Partially dependent ** **(*n* = 98)**	**Dependent ** **(*n* = 90)**		
Deciding what medication to take	2.15 ± 1.04	2.03 ± 0.93	2.13 ± 0.93	0.479	0.620
Taking medicine	2.72 ± 1.04	2.94 ± 1.16	2.89 ± 0.97	1.769	0.172
Exercising	2.76 ± 1.02	2.98 ± 1.03	2.97 ± 1.02	2.183	0.114
Calling doctors/911 (or 999 in Hong Kong)	3.24 ± 0.93	3.29 ± 0.81	3.22 ± 0.83	0.140	0.870
Being reminded to take medicine	3.66 ± 0.77	3.51 ± 0.83	3.30 ± 0.97	6.010	0.003

^a^*SD* Standard deviation

#### Chores category

The three groups preferred robots to assist in majority (19 out of 21) of the tasks in the chores category. However, the three groups preferred humans to assist in preparing meals/cooking. The independent participants preferred humans to assist in setting the table. One-way ANOVA did not reveal any significant difference in preference between participants with different dependence levels (Table 5).

**Table 5:** Assistance preferences on domestic tasks (under the chores category) from robots in older adults with different functional dependence level

**Domestic task**	**Preferences (Mean** **±** **SD**^**a**^**)**			***F***	***p***
	**Independent ** **(*n* = 197)**	**Partially dependent ** **(*n* = 98)**	**Dependent ** **(*n* = 90)**		
Preparing meals/cooking	2.52 ± 1.01	2.78 ± 1.05	2.98 ± 1.08	6.366	0.002
Setting the table	2.95 ± 0.92	3.23 ± 0.81	3.27 ± 0.80	5.653	0.004
Grocery shopping	3.14 ± 0.94	3.45 ± 0.89	3.37 ± 0.88	4.308	0.014
Repairing plumbing	3.37 ± 0.90	3.65 ± 0.81	3.69 ± 0.80	6.109	0.002
Washing dishes by hand	3.17 ± 1.01	3.18 ± 1.05	3.40 ± 0.95	1.719	0.181
Keeping refrigerator clean/stocked	3.35 ± 0.90	3.54 ± 0.78	3.46 ± 0.80	1.745	0.176
Doing laundry	3.62 ± 0.83	3.60 ± 0.86	3.83 ± 0.59	2.638	0.073
Painting	3.51 ± 0.80	3.67 ± 0.74	3.58 ± 0.73	1.530	0.218
Watering plants	3.19 ± 0.85	3.43 ± 0.79	3.28 ± 0.82	2.791	0.063
Sorting mail, shredding, throwing away junk mail	3.14 ± 0.97	3.52 ± 0.97	3.40 ± 0.78	6.154	0.002
Gardening/pruning	3.19 ± 0.84	3.32 ± 0.77	3.18 ± 0.80	0.955	0.386
Loading/unloading dishwasher	3.63 ± 0.67	3.56 ± 0.72	3.64 ± 0.61	0.485	0.616
Taking out trash/recyclables	3.65 ± 0.69	3.59 ± 0.82	3.60 ± 0.78	0.303	0.739
Making bed/changing sheets	3.39 ± 0.85	3.65 ± 0.78	3.52 ± 0.86	3.308	0.038
Changing light bulbs	3.57 ± 0.85	3.72 ± 0.85	3.72 ± 0.84	1.517	0.221
Cleaning bathrooms	3.63 ± 0.78	3.72 ± 0.80	3.79 ± 0.74	1.424	0.242
Cleaning windows	3.69 ± 0.72	3.87 ± 0.53	3.80 ± 0.72	2.449	0.088
Sweeping/scrubbing/mopping	3.64 ± 0.75	3.80 ± 0.72	3.86 ± 0.61	3.257	0.040
Controlling for pests/rodents	3.43 ± 0.86	3.60 ± 0.78	3.68 ± 0.78	3.227	0.041
Cleaning kitchen	3.64 ± 0.79	3.81 ± 0.74	3.82 ± 0.70	2.571	0.078
Maintaining lawn/raking leaves	3.56 ± 0.68	3.44 ± 0.68	3.42 ± 0.64	1.765	0.173

^a^*SD* Standard deviation

#### Information management category

The three groups preferred robots to assist in all the tasks in the information management category. One-way ANOVA did not reveal any significant difference in preference between participants with different functional dependence levels (Table 6).

**Table 6:** Assistance preferences on domestic tasks (under the information management category) from robots in older adults with different functional dependence level

**Domestic task**	**Preferences (Mean** **±** **SD**^**a**^**)**			***F***	***p***
	**Independent ** **(*n* = 197)**	**Partially dependent ** **(**n** = 98)**	**Dependent ** **(*n* = 90)**		
Getting information on weather/news	3.50 ± 0.79	3.51 ± 0.79	3.52 ± 0.77	0.019	0.981
Being reminded of appointments	3.61 ± 0.79	3.51 ± 0.86	3.44 ± 0.94	1.284	0.278
Being reminded of daily activities	3.52 ± 0.82	3.26 ± 0.95	3.57 ± 0.84	4.031	0.019
Monitoring home/warning about dangers	3.57 ± 0.86	3.76 ± 0.98	3.76 ± 0.76	2.115	0.122

^a^*SD* Standard deviation

#### Manipulating objects category

The three groups preferred robot to assist in all the tasks in the manipulating objects category. One-way ANOVA did not reveal any significant difference in preference between participants with different functional dependence levels (Table 7).

**Table 7:** Assistance preferences on domestic tasks (under the manipulating objects category) from robots in older adults with different functional dependence level

**Domestic task**	**Preferences (Mean** **±** **SD**^**a**^**)**			***F***	***p***
	**Independent ** **(*n* = 197)**	**Partially dependent ** **(*n* = 98)**	**Dependent ** **(*n* = 90)**		
Opening and closing doors/drawers	3.56 ± 0.70	3.58 ± 0.66	3.70 ± 0.61	1.418	0.244
Finding/delivering items	3.51 ± 0.81	3.71 ± 0.69	3.74 ± 0.63	4.350	0.014
Reaching for objects	3.57 ± 0.75	3.47 ± 0.82	3.72 ± 0.62	2.786	0.063
Fetching objects from floor or other room	3.56 ± 0.73	3.70 ± 0.72	3.70 ± 0.64	1.979	0.140
Picking up/moving heavy objects	3.75 ± 0.67	3.87 ± 0.70	3.83 ± 0.69	1.200	0.302

^a^*SD* Standard deviation

## Discussion

This study makes a novel attempt and compares the preferences of older adults with different functional dependence levels. Our findings report the specific preferences of older adults with different functional dependence levels. They have varying preferences for assistance in personal care and leisure activities but have similar preferences for assistance in health, chores, information management, and manipulating objects.

### Differences in preferences between older adults with different functional dependence levels

Independent participants exhibited a significant preference for human assistance in tasks under the personal care category when compared to partially dependent and dependent participants (*p* < 0.001; effect size: medium to large). While the *p* values confirmed statistical significance, the substantial effect sizes further highlighted the practical significance of the research findings. Independent participants preferred humans over robots to perform personal care tasks, including shaving, bathing, washing/combing hair, eating/feeding myself, getting dressed, walking, and brushing teeth (mean < 3). Generally, tasks on personal care involve delicate physical touch and close interaction. Humans are biological bodies and are perceived to be gentle and better able to carry out delicate personal care. Robots are computer products and perceived as rude. They may cause uncomfortable feelings throughout the processes. Consistently, robots are considered less useful in performing personal care-related tasks [1, 13, 14].

Another plausible explanation could be related to the Chinese belief system prevalent among older adults in Hong Kong. Filial piety is an essential aspect of Chinese culture. It emphasizes kindness and deep respect towards one’s parents. Older adults are often held in high regard and usually receive care from their family caregivers [24, 25]. They do not expect robots to take up the traditional caregiving roles within the family. Additionally, maintaining face or preserving dignity is highly valued in Chinese culture. Losing face can often result in feelings of shame [24, 25]. Older adults may perceive a loss of face and feel shame when relying heavily on robots for assistance with personal care tasks.

In contrast to the existing understanding, our findings depicted that the partially dependent and dependent participants preferred robots to assist in personal care, such as bathing, walking, and brushing teeth. Owing to the prevalence of nuclear families in Hong Kong, a growing number of older adults are residing separately from their younger generations. While a significant proportion of disabled older adults are unable to obtain the necessary practical assistance [2], we speculate that the partially dependent and dependent participants do not have adequate human resources to assist in personal care at the moment. Choosing a robot to assist in personal care appears to be an option.

Under the leisure activities category, the dependent participants exhibited a significantly less favorable preference for human assistance in calling family/friends compared to independent participants. Unlike independent older adults, who can travel freely and maintain a larger social circle, dependent older adults frequently have limited opportunities to engage in leisure activities and have a smaller, or even very small, social circle. They may have fewer expectations of human assistance, but still desire robots to connect them with others, particularly their family and friends.

Partially dependent and dependent participants reported a higher mean score than the independent ones in many domestic tasks under the health, chores, information management, and manipulating objects categories. However, the differences were statistically non-significant. Older adults’ preferences may become more obvious over time when they have direct interactions with the robots and/or when the robots are better developed. Thus, comparison of research findings over an extended period can detect the changes in older adults’ preferences over time.

### Similarities in preferences in older adults with different functional dependence levels

Participants, regardless of functional dependence level, preferred humans to assist in preparing meals/cooking, the only chore that was preferred to be assisted by humans. Chinese culture places a high value on the proper preparation and cooking of meals. Chinese cuisine is well-known for its rich flavors, thoughtful food combination, complicated cooking techniques, diverse styles, and specific serving order. Chinese people believe that preparing meals properly and consuming nutritious foods are vital for health maintenance [26–28]. Given that robots are programed to operate in a standardized manner, they may be less preferred to assist with meal preparation and cooking, particularly for Chinese older adults whose cooking practices are deeply rooted in Chinese culinary traditions.

Participants preferred humans to assist in eating/feeding, the only domestic task under the personal care category that was preferred to be assisted by humans. Overall, older adults consider feeding involves human-like spoon feeding motion, such as tilting and retracting [29]. Possibly, they feel more comfortable to have humans over robots to assist in feeding.

Participants unanimously preferred humans to assist in domestic tasks under the leisure activities category, such as entertaining guests and being entertained. These tasks typically require communication and interaction. Humans are perceived as more creative and energetic than robots. These qualities enable humans to develop a more constructive and meaningful conversation with the older adults. Moreover, Chinese culture values interdependence and social connections. Older adults often rely on family bonds and community networks for support [24, 25]. The preference for human assistance may stem from the desire to maintain social connections with other people.

Moreover, participants with different functional dependence levels preferred humans to assist in deciding what medication to take, taking medicine, and walking. They may believe robots are not liable to the adverse consequences of any inappropriate decision. With more trust on humans, older adults tend to seek human effort to make medication-related decision [30]. The findings highlighted the critical role of human labor, particularly trained labor, in assisting older adults with critical health and medication issues that are unlikely to be completely replaced by robots.

However, participants, regardless of functional dependence level, preferred robots to assist in calling doctors/911 and being reminded to take medicine. Because robots can be programed to carry out a task at a specific time or condition, they are particularly helpful in providing instance and on-time notifications [31].

Participants preferred robots to assist the domestic tasks under the information management category. Older adults grew up in an era with no computers. Their ability in managing electronic information is considerably weak. Meanwhile, robots are computer products that can provide accurate, up-to-date, and comprehensive information while also sending reminders [31]. Robots can effectively overcome the limitations of older adults in using computer technology and provide practical benefits.

Participants unanimously preferred robots to assist in obtaining information on hobbies/topics of interest. Their responses are understandable, as robots are skillful in retrieving and processing large amounts of data.

Participants also preferred robots to assist in majority of the chore tasks. Older adults generally have reduced ability to perform chores, especially the physically demanding ones. Robots are thought to be stronger and more durable than humans when performing physically demanding tasks [31]. When chores are performed by robots, a more efficient outcome can be obtained [32].

Lastly, participants preferred robots to assist the domestic tasks under the manipulating objects category. Tasks related to manipulating objects are commonly mechanical and do not require much decision making. Robots can be considered substantially competent in this aspect and their involvement is unlikely to produce adverse consequences.

### Strengths, limitations, and recommendations for future research

This study was the first to compare assistance preferences on domestic tasks from robots in older adults with different functional dependence levels. It employed an adequate sample size and used valid instruments to collect data. The findings indicate that assistance preferences differ significantly between older adults with different functional dependence levels, demonstrating a medium to large effect size. The findings advance our understanding in this particular field. Future research can continue to investigate why the older adults hold such preferences. As functional dependence level is an indicator of functional abilities and can be contributed by several health conditions, future research is encouraged to investigate the preferences of older adults with specific health conditions to expand our knowledge.

Several limitations existed in this study. This study adopted convenience sampling which was more likely to result in a biased sample. The present findings are relevant to the older adults who share similar characteristics with the study sample. Future studies are suggested to adopt probability sampling to strengthen the research method.

Furthermore, some of the tasks on the checklist, such as maintaining the lawn or raking leaves, loading or unloading the dishwasher, are uncommon in Hong Kong. It is possible that some participants lacked experience or knowledge of a particular task and were unable to express their preferences. However, since participants’ responses for these tasks did not exhibit any extreme or unusual patterns, it can be assumed that they were able to interpret the questions. In the future, it is suggested that the checklist include an answer option of “not applicable” to allow participants to precisely indicate their responses.

Moreover, this study did not show any robots to the participants. Participants indicated their preferences on the basis of their imagination of robotic technologies. Without a common reference, some variations may exist in participants’ imagination. Future research can investigate older adults’ preferences on specific types of robots to complement the present findings.

Lastly, this study recruited older adults who had no prior experience with using robots. Participants’ responses may be influenced by their individual perceptions, lack of experience, or even misconceptions [7]. To further augment the present findings, how robotic experience influences the preferences of the older adults in receiving robot assistance warrants further exploration. A combination of unstructured or semi-structured interview in addition to the questionnaire is suggested. This approach can enhance our understanding of participants’ views and provide insights into their responses in the questionnaire.

### Implications for domestic care

The results obtained from this study have several implications for the use of robots as domestic assistants for older adults. Our findings indicate that assistance preferences on domestic tasks from robots vary with older adults’ functional dependence levels. Thus, the functional dependence level of the older adults must be assessed before the introduction of robots. This assessment ensures a good matching of the capabilities of the robots to the preferences of the older adults. Also, regular monitoring must be performed to detect changes in health conditions of the older adults and their acceptance of the robots. Thus, necessary adjustment can be made promptly.

Second, future development of robots for domestic care may be more aware of their capabilities in performing delicate physical care and communication which are important when assisting personal care and leisure activities for older adults. Older adults generally perceive robots are metallic, cold, and rude. Development of future robots may consider using other materials which can give the users a soft impression. Moreover, older adults generally perceive robots are weak in communication. In this regard, future robots should be able to adopt the appropriate language, methods of talking, and ways of responding which allow the older adults to emotionally interact with the robots [29].

Third, more efforts must be instilled to strengthen the capabilities of robots in carrying out domestic tasks for partially dependent and dependent older adults. Our findings indicate that these two categories of older adults are more open to robots for receiving assistant on the tasks under the personal care, leisure activities, chores, information management, and manipulating objects categories than independent older adults. As these two populations require a higher level and more varieties of assistance, the capabilities of the robots must be strengthened.

Fourth, it is reasonable to treat partially dependent and dependent older adults in a similar manner. Our research findings reveal that these two groups of older adults showed no significant difference in their assistance preferences on domestic tasks performed by robots. By providing partially dependent older adults with the same nature and level of robot assistance as their dependent counterparts, they can receive enhanced support and potentially delay functional decline. This approach may contribute to mitigating the progression of their dependency.

Fifth, an adequate level of human manpower must be retained to deliver health-related support to the older adults in various functional dependence levels. Our findings reveal that older adults generally are less open to robots for assistance on some tasks under the health category. Perhaps, combining the efforts of humans and robots may simultaneously bring benefits to older adults and human assistants. Ultimately, how this human–robot assistance model works must be determined.

## Conclusions

Aging commonly results in functional changes and increased functional dependence in certain older adults. These changes lead to an increased reliance on assistance for daily living. Robots have the potential to carry out various domestic tasks and can be considered as emerging sources of support to the community-dwelling older adults. Older adults with different functional dependence levels may have different preferences on receiving assistance from the robots. This study made a novel attempt in comparing their preferences.

Independent older adults demonstrated significant preference for human assistance, mostly in the domestic tasks under the personal care category. Concurrently, partially dependent and dependent older adults reported a significant lesser preference for human assistance or a higher preference for robot assistance in most of the domestic tasks under the personal care category. Meanwhile, older adults, regardless of functional dependence levels, revealed similar preferences in the domestic tasks under the leisure activities, health, chores, information management, and manipulating objects categories.

This study recommends robot designers and care givers to consider older adults’ specific preferences before designing and adopting robots in domestic care. The capabilities of the robots must be strengthened to carry out the tasks that are preferred to be assisted by robots, especially for partially dependent and dependent older adults who require a higher level and a wider range of capabilities from robots. Combining human and robot efforts may be required to complete tasks that are less desirable to be assisted solely by robots. This human–robot assistance model may bring benefits to all the parties and deserves further investigation.

## Data Availability

The data used in this study are available from the corresponding author on reasonable request. The use of the Katz Index of ADL was approved by the Gerontological Society of America. The use of the Assistance Preference Checklist was granted by Prof. Cory-Ann Smarr.

## Abbreviations

Katz ADL: Katz Index of Independence in Activities of Daily Living
ANOVA: Analysis of variance
